# PD1+ TIGIT+ CD4+ T cells predict response to anti-TNF in rheumatoid arthritis and spondyloarthritis

**DOI:** 10.1136/rmdopen-2025-006537

**Published:** 2026-05-20

**Authors:** Samuel Bitoun, Matthieu Roulleaux-Dugage, Marie Naigeon, Caroline de Oliveira, Caroline Berthot, Xavier Mariette, Gaetane Nocturne, Nathalie Chaput

**Affiliations:** 1 Rheumatology Department, Université Paris-Saclay, Institut National de la Santé et de la Recherche Médicale (INSERM) UMR1184, Hôpital Bicêtre, Assistance Publique-Hôpitaux de Paris (APHP), CEA, FHU CARE 2, Le Kremlin-Bicêtre, France; 2 Laboratoire d’Immunomonitoing en Oncologie US23, Biothérapies Innovantes U1363, Université Paris-Saclay, Gustave Roussy, INSERM, Villejuif, France; 3 Département d’Innovation Thérapeutique et d’Essais Précoces (DITEP), Université Paris-Saclay, Gustave Roussy, Villejuif, France

**Keywords:** T Cells, Rheumatoid Arthritis, Spondyloarthritis, Anti-TNF

## Abstract

**Background:**

Programmed cell death protein 1 (PD-1) and T cell immunoreceptor with Ig and ITIM domains (TIGIT) are immune checkpoints expressed on T cells. Despite recent development of PD-1 agonists in rheumatoid arthritis (RA), little is known about PD-1 and TIGIT coexpression in RA and other immune-mediated inflammatory diseases (IMIDs). This study quantified PD-1 and TIGIT expression in CD4 and CD8 T cells in patients with IMID and examined associations with disease characteristics and anti-tumour necrosis factor (TNF) response.

**Methods:**

Biologics-naïve patients with RA and spondyloarthritis (AS) were followed prospectively to assess anti-TNF response at 12 months. Sjögren’s disease (SjD) patients and healthy volunteers (HV) were also included at baseline. PD-1 and TIGIT expression on CD4 and CD8 T cells was analysed by flow cytometry at baseline and after 3 months of anti-TNF treatment. Plasma cytokines were quantified using the Meso Scale Discovery assay.

**Results:**

67 patients were included. Median CD8^+^PD-1^+^TIGIT^+^ levels were higher in RA (24%) and SjD (23.2%) than in HV (18.8%) and AS (16.2%). CD4^+^PD-1^+^TIGIT^+^ were positively correlated with circulating immunoglobulin G (r=0.698, p<0.001), interferon gamma (r=0.635, p=0.017) and IP-10 (r=0.612 p=0.005) levels in patients with SjD. Baseline CD4^+^PD-1^+^TIGIT^+^ levels were higher in future anti-TNF responders (9.9%) compared with non-responders (6.1%, p=0.003). Both CD4^+^PD-1^+^TIGIT^+^ and CD8^+^PD-1^+^TIGIT^+^ cells increased after 3 months of anti-TNF treatment in RA and AS.

**Conclusion:**

CD8^+^PD-1^+^TIGIT^+^ T-cell expression is elevated in SjD and RA compared with HV and AS. CD4^+^PD-1^+^TIGIT^+^ levels are higher in future anti-TNF responders compared with non-responders and rise after treatment in patients with RA and AS. Further studies should clarify the functional role of CD4^+^PD-1^+^TIGIT^+^ cells in IMIDs.

**WHAT IS ALREADY KNOWN ON THIS TOPIC**

- PD-1 expression and TIGIT are checkpoint molecules regulating T-cell activation. There are limited studies in spondyloarthritis (AS) and Sjögren’s disease (SjD) on these markers.
- The dynamics of PD-1 and TIGIT had not been prospectively studied at the initiation and after anti-TNF in rheumatoid arthritis (RA) and AS.

**WHAT THIS STUDY ADDS**

- CD8^+^PD-1^+^TIGIT^+^ T cells are increased in patients with RA and SjD versus controls but not in patients with AS.
- CD4^+^PD-1^+^TIGIT^+^ T cells are positively associated with immunoglobulin G levels and circulating levels of interferon gamma in SjD.
- CD4^+^PD-1^+^TIGIT^+^ T cells are more frequent in future anti-TNF responders.

**HOW THIS STUDY MIGHT AFFECT RESEARCH, PRACTICE OR POLICY**

- CD4^+^PD-1^+^TIGIT^+^ elevation at baseline could be a predictive marker of response to anti-TNF.

## Introduction

 Immune checkpoint molecules such as PD-1 and Cytotoxic T-Lymphocyte-Associated protein-4 (CTLA-4) play an important role in the regulation of the activation of the immune system. These markers are upregulated on T-cell activation, but sustained high expression is associated with T-cell exhaustion.^1^ Engagement of PD-1 and CTLA-4 with their ligands results in the transmission of inhibitory signals, thereby limiting T-cell activation. They can be antagonised by anti-CTLA-4 and anti-PD-1/PD-L1 antibodies called immune checkpoint inhibitors, which have become a key treatment of many cancers even in the neoadjuvant setting.^2^ Before their use in oncology, where immune checkpoints are blocked to restore T-cell activation, they had already been targeted in autoimmune diseases with agonists such as abatacept, a CTLA-4-Ig agonist used in rheumatoid arthritis (RA), to reinforce inhibitory signalling.^3^ Recent work has shown the promising efficacy of PD-1 agonists tested in phase II studies in RA.^4^ High levels of PD-1 expression have been identified in T peripheral helper (Tph) cells. These cells, characterised as CD4^+^ PD-1^hi^ CXCR5^−^, are found in the peripheral blood and joints of patients with RA, where they are linked to disease activity.^5^ Tph cells were described as non-exhausted. They promote plasma cell differentiation via interleukin-21 (IL-21) and Signalling Lymphocytic Activation Molecule Family member 5 interactions.^5^

TIGIT is an inhibitory receptor expressed on T cells. Through binding to the poliovirus receptor, it not only directly dampens T-cell activation but also promotes the induction of tolerogenic dendritic cells, further contributing to immune suppression.^6^ TIGIT is expressed by effector T cells, where it decreases their activation in the cancer setting^7^ but also on regulatory T cells, where it leads to enhanced suppressive capacities.^8^ In the context of immune-mediated inflammatory disease (IMIDs), TIGIT overexpression on T cells is associated with less severe collagen-induced arthritis, a mouse model of RA.^9^ Moreover, recent evidence has highlighted the regulatory potential of citrullinated vimentin-reactive CD4^+^ CD39^+^ TIGIT^hi^ cells, which expanded in patients with drug-free remission RA.^10^ The expression of multiple inhibitory markers is a hallmark of regulatory cells.

Dual blockade of PD-1 and TIGIT leads to optimised CD8^+^ activation in lung cancer, further highlighting non-redundant pathways.^11^ Since PD-1^+^ TIGIT^+^ T cells are tumour-antigen specific in melanoma,^12^ we hypothesised that in IMIDs, this population could also be autoantigen specific, making it a key target for advancing our understanding of these diseases. Recent evidence highlighting the antigen-specific regulatory potential of CD4^+^CD39^+^TIGIT^hi^ cells further reinforces this hypothesis.^10^ While PD-1^hi^ Tph cells have been extensively studied, there are limited data on PD-1^+^ TIGIT^+^ T cells in IMIDs, their association with disease characteristics and activity,^13^ and their dynamics following treatment. Finally, CD4+PD-1+ cells are 9.5 times more likely to express TIGIT compared with CD4+PD1 cells in a transcriptome analysis of patients with RA.^14^ This study aimed to prospectively investigate the coexpression of PD-1 and TIGIT on blood CD4^+^ and CD8^+^ T cells in two autoimmune diseases, RA and Sjögren’s disease (SjD), a systemic autoimmune disorder, as well as in spondyloarthritis (AS), an inflammatory rheumatic disease. The primary objective of this work was to compare the expression of PD-1 and TIGIT between healthy volunteers (HV) and patients with IMID and between diseases. Second, we investigate how circulating cytokines may be associated with PD-1 and TIGIT. Finally, we examined the impact of this population on response to anti-TNF treatment in patients with RA and AS.

## Patients and methods

### Patients

Patients with RA, AS and SjD were recruited between February 2021 and November 2022 in the rheumatology department of Hôpital Bicêtre, an academic tertiary public hospital setting in France, a national and European reference centre for inflammatory arthritis and systemic autoimmune diseases.

Patients with RA fulfilled the American College of Rheumatology/European Alliance of Associations for Rheumatology (ACR/EULAR) 2010 criteria,^15^ patients with AS fulfilled the Assessment of SpondyloArthritis International Society criteria,^16^ and patients with SjD fulfilled the ACR/EULAR 2016 criteria.^17^ Patients were included if they were aged 18 or older, and for patients with RA and AS, anti-TNF naïve with a physician-planned anti-TNF initiation. They were treated according to local guidelines.

### Clinical and biological assessment

Comprehensive clinical and biological data for each patient were collected prospectively, including demographic information, age, disease duration (except for Sjögren’s), C reactive protein (CRP), previous and current treatments, Rheumatoid factor (RF), anti–cyclic citrullinated peptide (anti-CCP) antibodies, disease activity Disease Activity Score using 28 joints for RA and Bath Ankylosing Spondylitis Disease Activity Index for AS, and response to treatment.

Response to treatment was defined by treatment maintenance at 12 months post-treatment initiation. Patients who switched drugs due to side effects were excluded from this analysis. Since patient with RA and AS were both treated with anti-TNF, we decided to analyse the factors predicting response to treatment in both diseases at the same time.

### PD-1 and TIGIT markers assessment

Blood was collected at baseline and 3 months after treatment initiation for RA and AS. Patients with SjD were collected regardless of their current treatment and only once, as there is no approved drug for this disease. Heparin tubes were used for fresh whole-blood immune phenotyping. For surface staining, 100 µL of blood was incubated for 20 min at room temperature in the dark with liquid antibodies (panel, online supplemental table 1). Erythrocyte lysis was performed by adding 1 mL of Versalyse (Beckman) containing 25 µL of Fixative Solution (Beckman) for 20 min at room temperature in the dark. After two washes with 3 mL of 1× phosphate-buffered saline (PBS), cells were resuspended in 250 µL of 1× PBS. Stained cells were acquired using a Gallios flow cytometer (Beckman Coulter) and analysed using Kaluza Analysis software (Beckman Coulter).

The gating strategy of T cells expressing PD-1^+^ TIGIT^+^ CD4^+^ or CD8^+^ cells is detailed in online supplemental figure 1.

### Plasmatic dosages

#### Plasma preparation

Plasma was extracted from blood in a heparinised tube, by centrifugation at 2800 revolutions per minute (rpm) for 15 min at 15°C. A second centrifugation was made (5000 rpm, 20 min, 4°C) to remove residual platelets. The samples were stored at −80°C for later protein quantification.

#### Detection and quantification of plasmatic cytokines

All plasma samples were thawed and centrifuged at 5000 rpm for 20 min at 4°C to remove residual platelets. Cytokines and soluble proteins were measured using the Meso Scale Discovery (MSD) immunoassay (Rockville, Maryland, USA). Three MSD kits were used: ‘U-PLEX Biomarker Group 1 (hu)’ to measure IFN-β, IFN-λ1, IP-10 and IFN-γ; ‘U-PLEX Custom Biomarker (hu)’ to measure TNF and IL-6; and ‘S-PLEX Human IFN-α2a’ to measure IFN-α2a. Assays were performed following the manufacturer’s protocol using MSD 96-well plates and recommended diluents. Plates were read by MESO QuickPlex SQ 120 (MSD) and raw data were analysed with the Discovery Workbench 4.0 software (MSD). All measurements were run in duplicate for calculation of the variation coefficient and mean values, allowing elimination of mean values with a coefficient of variation (CV)>20% and CV>30% for IFN-β. The lower limits of detection and sample dilutions are described in online supplemental table 2.

### Patients’ involvement

Patients were not involved in the conduct or design of this study.

### Statistical analysis

Comparison of continuous variables between groups was performed using the Mann-Whitney test or Kruskal-Wallis test. The Fisher test was used to analyse the association between categorical variables. Correlations between two continuous variables were performed using the Spearman test. For longitudinal analyses (before and during treatment), the Wilcoxon matched-pairs signed rank test was used. Statistical analyses and graphs were performed using R software V.4.3. Values are expressed as median (IQR).

## Results

### Patients

A total of 67 individuals diagnosed with IMIDs were included in this study, of which 25 were patients with RA, 22 were patients with AS (of whom 2 also had Crohn’s disease) and 20 were patients with SjD. Demographics and disease-specific features are detailed in table 1. Baseline T-cell phenotyping was carried out for all 67 IMIDs patients, leading to the identification of CD4^+^ PD-1^+^ TIGIT^+^ and CD8^+^ PD-1^+^ TIGIT^+^ T cells as illustrated in online supplemental figure 1.

**Table 1:** Patients’ characteristics

Covariate	Patients with IMID	Patients with RA	Patients with AS	Patients with SjD
Number	n=67	n=25	n=22	n=20
Gender				
Female, n (%)	52 (77.6)	23 (92)	10 (45.5)	19 (95)
Male, n (%)	15 (22.4)	2 (8)	12 (54.6)	1 (5)
Age				
Years (min;max)	52.8 (22;86.17)	61 (28;84)	35 (22;70)	53.9 (25.96;86.17)
Disease duration				
Years, median (min;max)	5 (0;50)	4 (0;35)	6.5 (0;50)	NA
Missing, n	22	2	0	20
RF positivity	NA	24 (96%)	NA	NA
ACPA positivity	NA	24 (96%)	NA	NA
AS characteristics				
Axial AS		NA	20 (90.9%)	NA
Psoriatic arthritis			2 (9.1%)	
Baseline corticosteroid treatment				
No, n (%)	48 (71.6)	9 (36)	22 (100)	17 (85)
Yes, n (%)	19 (28.4)	16 (64)	0 (0)	3 (15)
Baseline treatment with methotrexate				
No, n (%)	22 (47.8)	3 (12)	19 (90.5)	0 (0)
Yes, n (%)	24 (52.2)	22 (88)	2 (9.5)	0 (0)
Missing, n	21	0	1	20
Treatment with NSAIDs at baseline				
No, n (%)	38 (80.9)	25 (100)	13 (59.1)	0 (0)
Yes, n (%)	9 (19.2)	0 (0)	9 (40.1)	0 (0)
Missing, n	20	0	0	20
Baseline CRP (mg/L)				
Median (min;max)	6 (0;98)	5.5 (0;98)	8 (0;33)	NA
Missing, n	22	1	1	
Baseline disease activity				
High, n (%)		6 (24)		
Moderate, n (%)		14 (56)		
Low, n (%)		1 (4)		
Remission, n (%)		4 (16)		
High, n (%)			12 (85.7)	
Low, n (%)			2 (14.3)	
Missing, n (%)			8	
High, n (%)				3 (15)
Moderate, n (%)				7 (35)
Low, n (%)				10 (50)
Response to anti-TNF-α at 1 year				
Responders	23 (67.6%)	12 (75%)	11 (61.1%)	NA
Non-responders	7 (20.6%)	3 (18.8%)	4 (22.2%)	NA
Discontinued due to adverse events prior to endpoint	4 (11.8%)	1 (12.3%)	3 (16.7%)	NA
Missing	13	9	4	NA

ACPA, anti-citrullinated protein antibodies; anti-TNF-α, anti–tumour necrosis factor alpha therapies; AS, spondyloarthritis; CRP, C reactive protein; IMID, immune-mediated inflammatory disease; NSAIDs, nonsteroidal anti-inflammatory drugs; RA, rheumatoid arthritis; RF, rheumatoid factor; SjD, Sjögren’s disease.

### Patients with SjD and RA are characterised by an increased proportion of circulating CD4^+^ and CD8^+^ PD-1^+^ TIGIT^+^ cells

**Figure 1:**
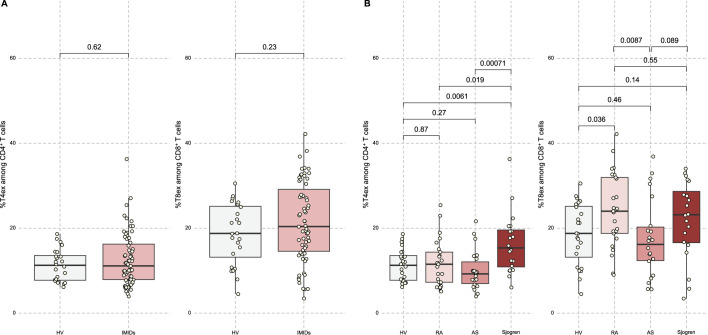
Patients with Sjögren’s disease (SjD) and rheumatoid arthritis (RA) harbour an increased proportion of PD-1^+^ TIGIT^+^ CD4^+^ T cells and CD8^+^ T cells (T4ex among CD4^+^ T cells and T8ex among CD8^+^ T cells), respectively. (A) Proportion of PD-1^+^ TIGIT^+^ CD4^+^ and CD8^+^ T cells depending on the presence (n=67) or absence (n=25) of an IMID. (B) Comparison between the proportion of PD-1^+^ TIGIT^+^ CD4^+^ and CD8^+^ T cells in HV (n=25) and each IMID cohort (RA: n=25, spondyloarthritis: n=22, SjD: n=20). AS, spondyloarthritis; HV, healthy volunteers; IMIDs, immune-mediated inflammatory diseases; RA, rheumatoid arthritis.

Overall, no increase in PD1^+^TIGIT^+^ T cells (CD4 or CD8) could be observed compared with HV when all IMIDs were analysed together (figure 1). Interestingly, CD4^+^PD1^+^TIGIT^+^ T cells were significantly and exclusively elevated in patients with SjD compared with HV and patients with RA or AS. For CD8^+^PD1^+^TIGIT^+^ T cells, a significant accumulation was observed in patients with RA with higher levels compared with patients with AS and HV. Thus, the accumulation of these PD1^+^TIGIT^+^ T-cell populations is not a common feature of IMIDs but rather a distinct characteristic of specific diseases.

### CD4^+^ PD-1^+^ TIGIT^+^ is associated with anti-SSA and anti-SSB positivity and with systemic IFN-γ-mediated inflammation in Sjögren disease

We then investigated if clinical and biological characteristics were associated with CD4^+^ and CD8^+^ PD-1^+^ TIGIT^+^. No relationship between CD4^+^ PD-1^+^ TIGIT^+^ or CD8^+^ PD-1^+^ TIGIT^+^ levels and disease activity scores could be observed for patients with RA, AS or SjD (online supplemental figure 2). Similarly, demographic characteristics such as age, sex, CRP or disease duration were not associated with CD4^+^ PD-1^+^ TIGIT^+^ or CD8^+^ PD-1^+^ TIGIT^+^ levels except age in all patients (r=0.34, p=0.005 online supplemental figure 3) driven by AS, where age was positively correlated with CD8^+^ PD-1^+^ TIGIT^+^ levels in an univariate analysis (r=0551, p=0.008 online supplemental figure 2) but not in a multivariate analysis (online supplemental table 3). CD4^+^ PD-1^+^ TIGIT^+^ or CD8^+^ PD-1^+^ TIGIT^+^ levels were not significantly different in patients with or without cotreatment with steroids or methotrexate (data not shown).

**Figure 2:**
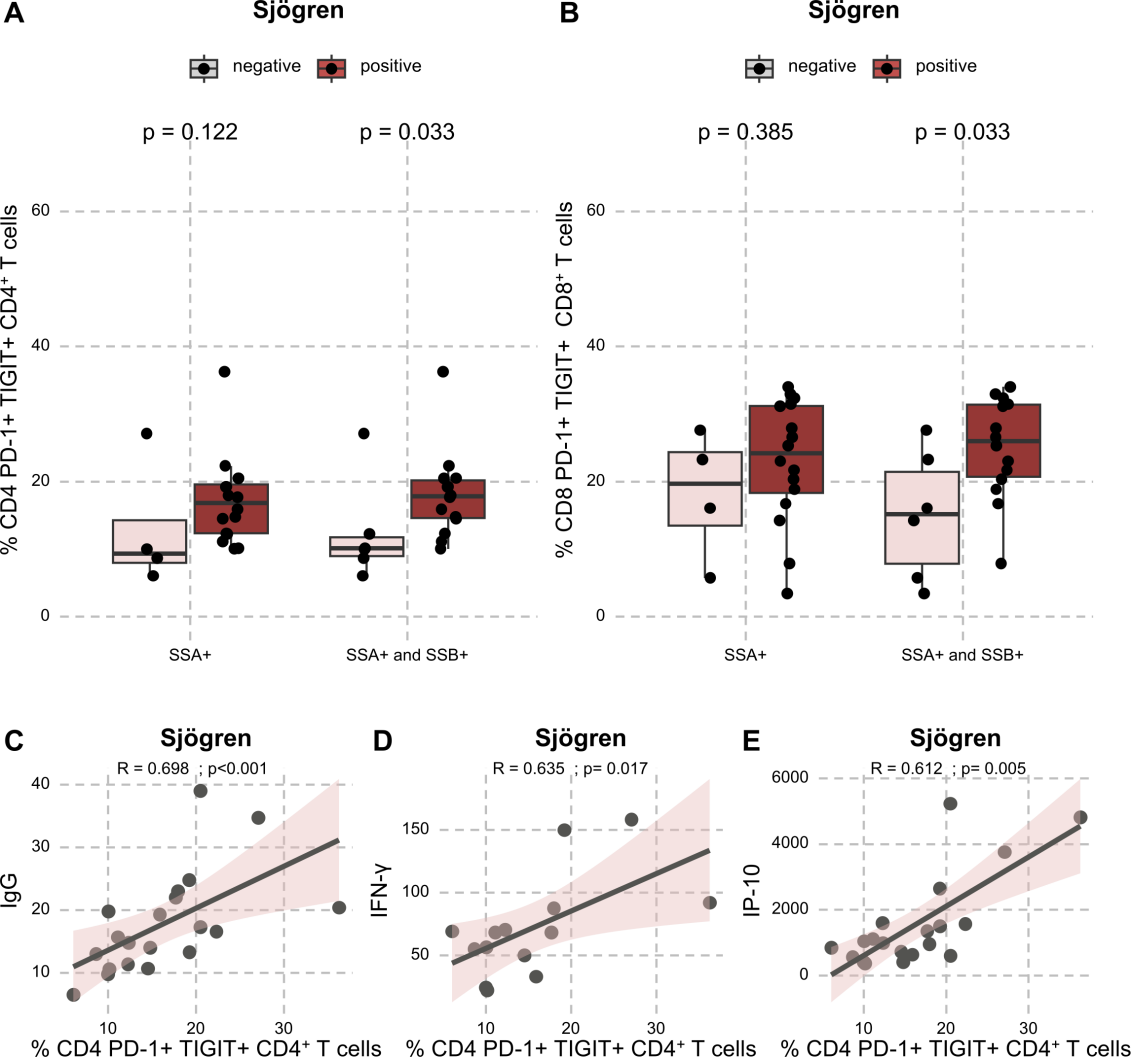
PD-1^+^ TIGIT^+^ CD4^+^ and CD8^+^ T cells association with disease parameters and cytokines in patients with Sjögren’s disease (SjD). (A) Proportion of PD-1^+^ TIGIT^+^ among CD4^+^ T cells depending on anti-SSA with or without anti-SSB positivity in patients with SjD (n=20). (B) Proportion of PD-1^+^ TIGIT^+^ among CD8^+^ T cells depending on anti-SSA with or without anti-SSB positivity in patients with SjD (n=20). (C) Correlation between the baseline proportion of PD-1^+^ TIGIT^+^ among CD4^+^ T cells and IgG levels (n=20), IFN-γ (n=14) (D) and IP-10 (n=20) (E) in patients with SjD. IgG, immunoglobulin G; IFN-γ, interferon gamma.

Given the accumulation of CD4^+^ PD-1^+^ TIGIT^+^ in patients with SjD, we further investigated other potential correlates in this population. Interestingly, patients with the double positivity of anti-SSA and anti-SSB antibodies harboured higher CD4^+^ PD-1^+^ TIGIT^+^ and CD8^+^ PD-1^+^ TIGIT^+^ levels (17.8% (IQR: 14.6–20.2) versus 10.1% (IQR: 9.0–11.8), p=0.033 for CD4^+^ PD-1^+^ TIGIT^+^ and 26.0% (IQR: 20.7–31.4) versus 15.2% (IQR: 7.9–21.5), p=0.033 for CD8^+^ PD-1^+^ TIGIT^+^) than patients with no autoantibody or with only anti-SSA antibodies (figure 2A,B).

In patients with SjD, interestingly, a positive correlation was observed between baseline CD4^+^ PD-1^+^ TIGIT^+^ proportions and immunoglobulin G (IgG) levels (R=0.698, p<0.001, figure 2C), which might give an insight into the association between this subset in hypergammaglobulinaemia. IFN-γ plasma levels (R=0.635, p=0.017, figure 2D) and IP-10 plasma levels (R=0.612, p=0.005, figure 2E) were also associated with baseline CD4^+^ PD-1^+^ TIGIT^+^ proportions. No other significant correlation was observed in other disease cohorts for CD4^+^ PD-1^+^ TIGIT^+^ (online supplemental figure 4).

Similarly, in the overall cohort as well as for each specific disease, baseline CD8^+^ PD-1^+^ TIGIT^+^ levels did not correlate with baseline CRP, IFN-λ1, IFN-α2a, IFN-β, IFN-γ, IL-6, IP-10 or TNF-α plasmatic concentrations (online supplemental figure 5).

### Baseline levels of CD4^+^ PD-1^+^ TIGIT^+^ T cells are higher in patients with RA and AS responding to anti-TNF compared with non-responders

We then investigated if the baseline proportions of CD4^+^ PD-1^+^ TIGIT^+^ or CD8^+^ PD-1^+^ TIGIT^+^ could be associated with response to anti-TNF in patients with RA and AS.

**Figure 3:**
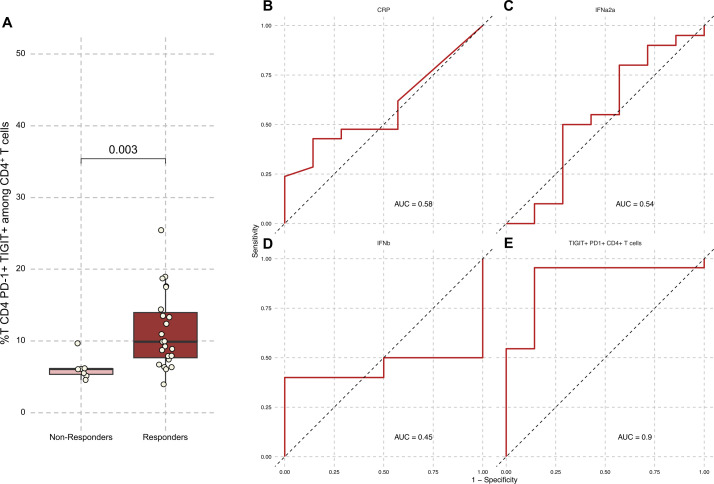
Patients with spondyloarthritis (AS) or rheumatoid arthritis (RA) responding to anti-TNF harbour an increased baseline proportion of PD-1^+^ TIGIT^+^ cells among CD4^+^ T cells. (A) Proportion of PD-1^+^ TIGIT^+^ among CD4^+^ T cells depending on response to anti-TNF treatment (n=7 non-responders, n=23 responders). ROC curves predicting response to anti-TNF treatment in patients with RA and AS of (B) CRP levels at baseline (C) IFNa2a levels at baseline (D) IFNb levels at baseline (E)% PD-1^+^TIGIT^+^ CD4 T cells among CD4 T cells at baseline. Responders: patients’ ongoing treatment at 12 months. Non-responders: patients who discontinued treatment for lack of efficacy before 12 months. anti–TNF, anti–tumour necrosis factor; AUC, area under the curve; CRP, C reactive protein; IFN-α2a, interferon alpha-2a; IFN-β, interferon beta; ROC, receiver operating characteristic; TIGIT, T cell immunoreceptor with Ig and ITIM domains.

Interestingly, percentages of baseline CD4^+^ PD-1^+^ TIGIT^+^ among CD4^+^ T cells were significantly higher in anti-TNF responders compared with non-responders (9.89% (7.64–14.0) vs 6.07% (5.34–6.14), respectively, p=0.003, online supplemental figure 6), suggesting that low baseline CD4^+^ PD-1^+^ TIGIT^+^ might specifically be associated with resistance to anti-TNF. Importantly, the percentage of CD8^+^ PD-1^+^ TIGIT^+^ was not significantly different in responders and non-responders to anti-TNF (19.44% (15.1–29.5) vs 19.4% (10.6–23.2), respectively, p=0.45, figure 3B). Circulating cytokines at baseline did not predict response to anti-TNF with a higher area under the curve (AUC) of the receiver operating characteristic (ROC) curve compared with CD4^+^ PD-1^+^ TIGIT^+^ (figure 3C,D) or compared between responders and non-responders (online supplemental figure 7). Finally, CD4^+^PD-1^+^ (p=0.0065) and CD4^+^TIGIT^+^ (p=0.023) were also significantly higher in future responders compared with non-responders (online supplemental figure 8). However, all other biomarkers performed less well than CD4^+^ PD-1^+^ TIGIT^+^ cells, which showed the highest predictive performance with an AUC of 0.90 (figure 3E and online supplemental figure 9). These results suggest that a high baseline level of circulating CD4^+^ PD-1^+^ TIGIT^+^ cells may predict response to anti-TNF therapy in patients with RA and AS.

### Dynamics of PD-1^+^ TIGIT^+^ T cells treated with anti-TNF therapy in patients with RA and AS

**Figure 4:**
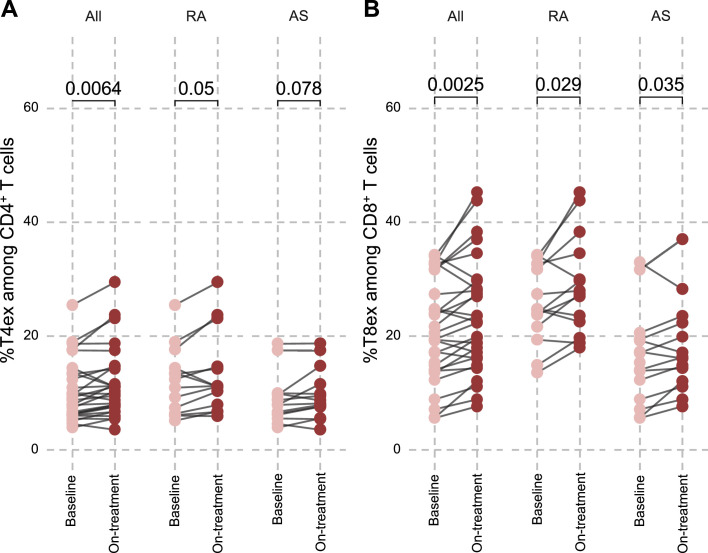
Patients with RA or spondyloarthritis (AS) show an increased proportion of PD-1^+^ TIGIT^+^ cells among CD4^+^ and CD8^+^ T cells after 3 months of treatment with anti-TNF. (A) Paired comparison of the proportion of PD-1^+^ TIGIT^+^ among CD4^+^ T cells before and after anti-TNF in AS (n=15), RA (n=15) and all patients (n=30). (B) Paired comparison of the proportion of PD-1^+^ TIGIT^+^ among CD8^+^ T cells before and after anti-TNF in AS (n=15), RA (n=15) and all patients (n=30). anti–TNF, anti–tumour necrosis factor; AS, spondyloarthritis; RA, rheumatoid arthritis.

We next analysed longitudinal changes in PD-1^+^ TIGIT^+^ T cells following anti-TNF initiation in patients with RA (n=15) and AS (n=15). As shown in figure 4A, the frequency of CD4^+^ PD-1^+^ TIGIT^+^ T cells increased significantly after 3 months of treatment in the overall cohort (p=0.0064). A similar trend was observed within disease subgroups (p=0.05 in patients with RA, p=0.078 in patients with AS). For CD8^+^ T cells, PD-1^+^ TIGIT^+^ frequencies also increased after anti-TNF initiation. The effect was significant in the global cohort (p=0.0025) and remained significant in subgroups (p=0.029 in patients with RA, p=0.035 in patients with AS) (figure 4B). These results demonstrate that anti-TNF therapy is consistently associated with an expansion of PD-1^+^ TIGIT^+^ T cells, affecting both CD4^+^ and CD8^+^ compartments.

## Discussion

This study highlights the role of PD-1^+^ TIGIT^+^ T cells in IMIDs, with significant association with both disease parameters and therapeutic responses. While these cells are not universally elevated across all IMIDs, their accumulation in patients with SjD for CD4^+^ and patients with RA for CD8^+^ and correlation with key clinical and biological markers underscore their relevance as potential biomarkers and mediators of disease processes.

In patients with SjD, the accumulation of CD4^+^ PD-1^+^ TIGIT^+^ T cells appears to be a defining feature. The observed association between these cells and anti-SSA/SSB double positivity, as well as their correlation with systemic markers of inflammation (IgG, IFN-γ, IP-10), suggests a central role for these cells in mediating or reflecting immune activation in SjD. Elevated CD4^+^ PD-1^+^ TIGIT^+^ cells may reflect chronic immune activation driven by IFN-γ. These cells could represent an exhausted phenotype attempting to regulate excessive immune responses, or alternatively, a pathogenic population contributing to disease progression through the production of pro-inflammatory cytokines. Type 1 IFN, which is suspected to play a more important role than IFN-γ in SjD, is not associated with the percentage of CD4^+^PD-1^+^TIGIT^+^. Type I interferon has been shown to increase PD-1 but decrease TIGIT expression.^16^ This might explain the lack of association between type I IFN and CD4^+^ PD-1^+^ TIGIT^+^. Given the correlation with anti-SSA/SSB positivity and systemic inflammation, CD4^+^ PD-1^+^ TIGIT^+^ cells could serve as a biomarker for disease severity or activity in SjD. Indeed, double autoantibody positivity is a hallmark of a more severe endotype of patients with SjD with increased interstitial lung disease.^18^ The positive correlation between CD4^+^ PD-1^+^ TIGIT^+^ and IgG levels indicates a potential link between this T-cell subset and humoral immune responses, possibly reflecting an ongoing or heightened immune reaction. This observation suggests that these T lymphocytes could be ‘pathologically expanded peripheral T helper’ cells, as previously described, capable of promoting the differentiation of B lymphocytes into plasma cells.^5^ However, this study does not directly demonstrate it. While other studies have reported associations between Tfh and SSA and SSB autoantibodies, they did not observe the association with IgG.^19^ The correlations with IFN-γ and IP-10—both key mediators of inflammation—suggest that CD4^+^ PD-1^+^ TIGIT^+^ T cells may contribute to or be influenced by inflammatory pathways. This finding could imply that these cells serve as biomarkers for disease activity or immune dysregulation, warranting further investigation to confirm their role in pathogenesis or disease progression. However, this work did not identify an association between activity score (as reflected by EULAR Sjögren’s Syndrome Disease Activity Index) and PD-1^+^TIGIT^+^ T cells. This might be due to the low representation of severe patients (only 15%). Larger cohorts and longitudinal studies are needed to assess their predictive value in tracking disease progression or therapeutic response.

While SjD is characterised by increased levels of CD4^+^ PD-1^+^ TIGIT^+^ cells, distinct patterns emerge in patients with RA and AS. Increased CD8^+^ PD-1^+^ TIGIT^+^ T cells in patients with RA suggest a role for cytotoxic T cells in driving inflammation, potentially through TNF-α and IFN-γ pathways. To further understand the function of the CD8^+^TIGIT^+^PD-1^+^ subset, we hypothesise that some cells could be autoantigen specific. However, this study did not directly demonstrate it. TIGIT and PD-1 increase with antigenic stimulation, especially in cancer patients, where they are more likely to be tumour specific.^20^ This could explain the elevation of CD8^+^TIGIT^+^PD-1^+^ cells in patients with RA and not in patients with AS, a disease without antigen autoimmunity. However, CD4^+^ PD-1^+^ TIGIT^+^ levels were not significantly elevated compared with HV, suggesting that regulatory or helper T-cell populations may not be the primary contributors to inflammation in RA. The differential accumulation of PD-1^+^ TIGIT^+^ T cells underscores the heterogeneity of IMIDs, emphasising the need for disease-specific biomarkers.

CD4^+^PD-1^+^TIGIT^+^ cells might have regulatory properties in RA and AS. The regulatory properties of TIGIT in IMIDs have been suggested by the efficacy of agonistic TIGIT antibodies in mouse models of multiple sclerosis.^21^ Recent data also demonstrate that a subset of CD4^+^CD39^+^TIGIThi T cells, which are citrullinated vimentin specific and show a regulatory profile. This subset increases in patients with RA in drug-free remission.^10^ FoxP3^+^TIGIT^+^ Tregs can specifically suppress Th1 and Th17 cells but not Th2 responses.^8^ This might explain why, in our study, a high proportion of a potentially regulatory subset of CD4^+^TIGIT^+^PD-1^+^ cells at baseline is associated with anti-TNF response both in RA, a Th1-driven disease, and AS, a Th17-driven disease^22^. On the other hand, higher levels of these cells in responders compared with non-responders and HV suggest that CD4^+^PD-1^+^ TIGIT^+^ T cells may serve as a biomarker for TNF-α-dependent inflammation. CD4^+^PD-1^+^ TIGIT^+^ cells may reflect an inflammatory milieu driven by TNF-α, IFN-γ and related cytokines. Patients with lower baseline levels of these cells might rely on alternative inflammatory pathways (eg, IL-6 and IL-17) that are less responsive to anti-TNF-α therapy, explaining the observed resistance. Measuring baseline CD4^+^PD-1^+^ TIGIT^+^ levels could help stratify patients likely to respond to anti-TNF-α, enabling personalised treatment approaches. This could reduce unnecessary exposure to ineffective therapies and guide the use of alternative treatments, such as IL-6 or Janus kinase inhibitors, for non-responders. This observation reinforces the idea that CD4^+^PD-1^+^ TIGIT^+^ cells play a contextual role, acting as a potential biomarker of Th1-driven inflammation, particularly relevant in conditions where TNF-α is central. This double positive subset could be associated with a specific endotype inside each disease which requires more patients to be characterised.

While this study provides valuable insights, several limitations must be addressed. The lack of longitudinal data in patients with SjD limits the ability to assess causality or dynamic changes in PD-1^+^ TIGIT^+^ T cells during disease progression or treatment. Additional studies in other IMIDs, such as systemic lupus erythematosus or inflammatory bowel disease, could further elucidate the generalisability of these findings. Functional studies are needed to determine whether PD-1^+^ TIGIT^+^ T cells are primarily regulatory, exhausted or pathogenic in each disease context. This will help clarify their role in mediating therapeutic responses. Characterisation of T follicular helper, Tph cells using CXCR5 and regulatory T cells has not been performed in this study.

The increase of CD4 and CD8 PD-1^+^TIGIT^+^ cells 3 months after anti-TNF treatment initiation could reflect either reduced inflammation leading to T-cell rebalancing or, alternatively, compensatory regulatory expansion. Further work should require a more detailed phenotyping to conclude if these subsets are activated or exhausted. Interestingly, the in vitro use of agonistic anti-TIGIT antibody after T-cell stimulation can lead to suppression of Tfh cells and enhancement of the suppressive capacity of regulatory T cells, highlighting the complexity of this marker in AID.^21^

This study highlights the heterogeneity of PD-1^+^ TIGIT^+^ T-cell populations across IMIDs and their potential as biomarkers for disease-specific immune activation. In SjD, their accumulation is associated with systemic IFN-γ-driven inflammation, whereas in RA and AS, baseline CD4^+^ PD-1^+^ TIGIT^+^ levels predict response to anti-TNF-α therapy. These findings pave the way for further exploration of PD-1^+^ TIGIT^+^ T cells as both diagnostic and therapeutic targets in IMIDs.

## Supplementary material

10.1136/rmdopen-2025-006537 online supplemental file 1

## Data Availability

Data are available upon reasonable request.
