# Supplementary material for: PD1+ TIGIT+ CD4+ T cells predict response to anti-TNF in rheumatoid arthritis and spondyloarthritis
Source: RMD Open. 2026 May 20;12(2):e006537. doi: 10.1136/rmdopen-2025-006537 (PMC13201991; doi:10.1136/rmdopen-2025-006537)
Supplement: online supplemental file 1 [file rmdopen-12-2-s001.docx]

**Supplementary Table 1:**

Antibodies used in the flow cytometry panel

| **Target** | **Fluorochrome** | **Provider** | **Dilution** | **Clone** | **Reference** |
| --- | --- | --- | --- | --- | --- |
| CD3 | AA750 | Beckman Coulter | 1:20 | UCHT1 | A94680 |
| CD4 | AA700 | Beckman Coulter | 1:20 | B10824 | 13B8.2 |
| CD56 | APC | Miltenyi Biotec | 1:50 | REA196 | 130-113-310 |
| CD8 | VioGreen | Miltenyi Biotec | 1:50 | REA734 | 130-110-684 |
| PD-1 | PerCP-cyanine 5.5 | Beckman Coulter | 1:10 | PD1.3 | B36123 |
| TIGIT | PE | Miltenyi | 1:25 | REA1004 | 130-116-814 |
| TIGIT | PE | eBioscience | 1:20 | MBSA43 | 12-9500-42 |

**Supplementary Table 2:**

**Plasma cytokine and soluble protein assays.** The reference of the kits and sample dilutions are given for each soluble protein. LLOD: lower limit of detection

| **Target soluble protein** | **Provider** | **Kit** | **Catalog number** | **Sample dilution** | **LLOD** |
| --- | --- | --- | --- | --- | --- |
| IFN-β | MSD | U-PLEX Biomarker Group 1 (hu) | K15067L-1 | 1:1 | 3.1 pg/ml |
| IFN-λ1 | MSD |  |  |  | 1.2 pg/ml |
| IFN-γ | MSD |  |  |  | 1.7 pg.ml |
| IP-10 | MSD |  |  |  | 0.49 pg/ml |
| TNF | MSD | U-PLEX Custom Biomarker (hu) | K15067M-1 | 1:1 | 0.51 pg/ml |
| IL-6 | MSD |  |  |  | 0.33 pg/ml |
| IFN-α2a | MSD | S-PLEX Human IFN-α2a | K151P3S-1 | 1:1 | 4.9 fg/ml |


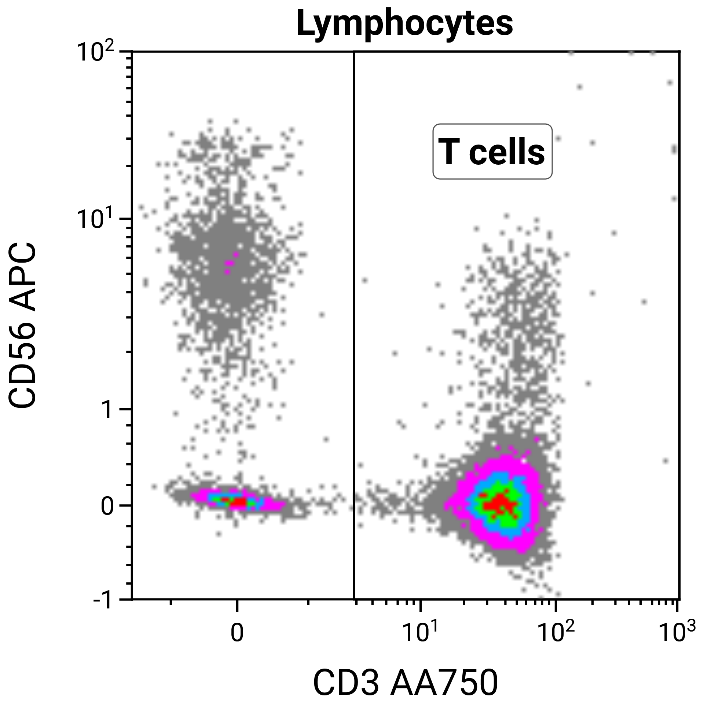

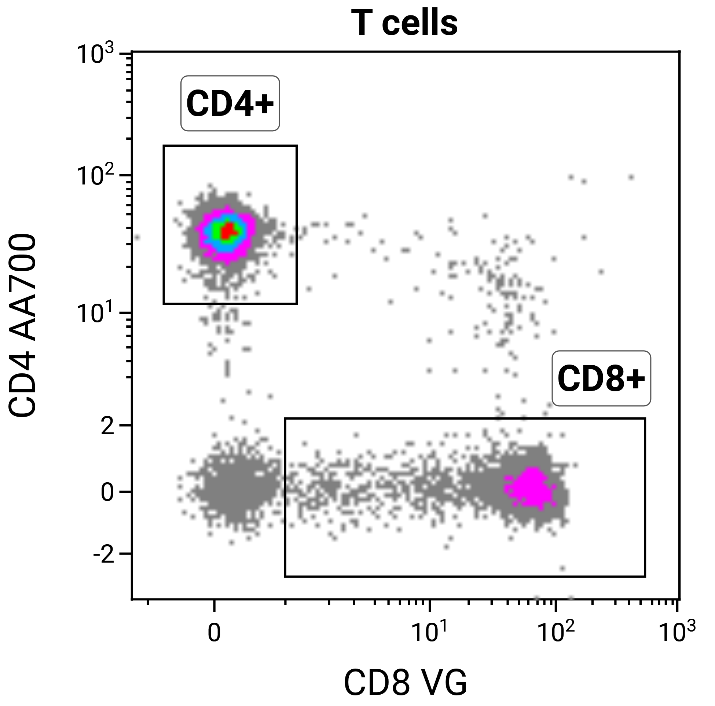

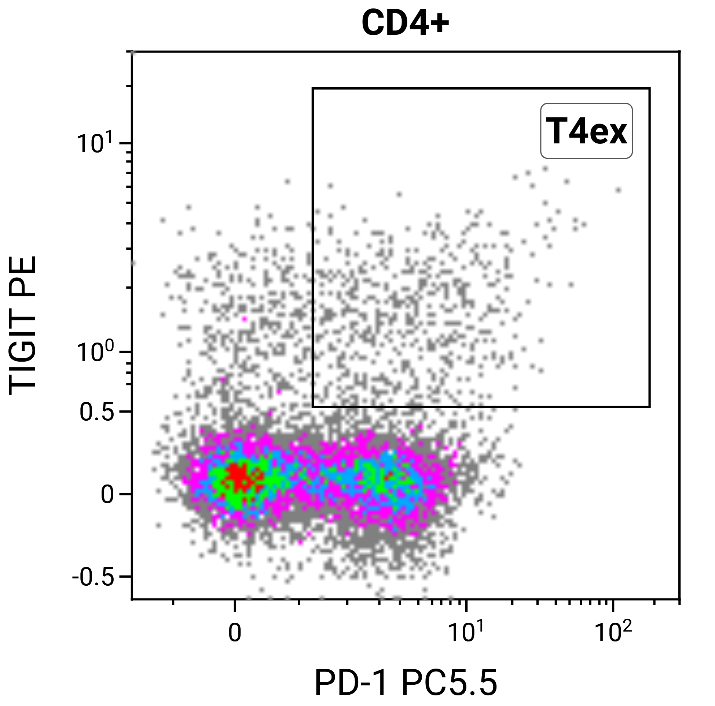

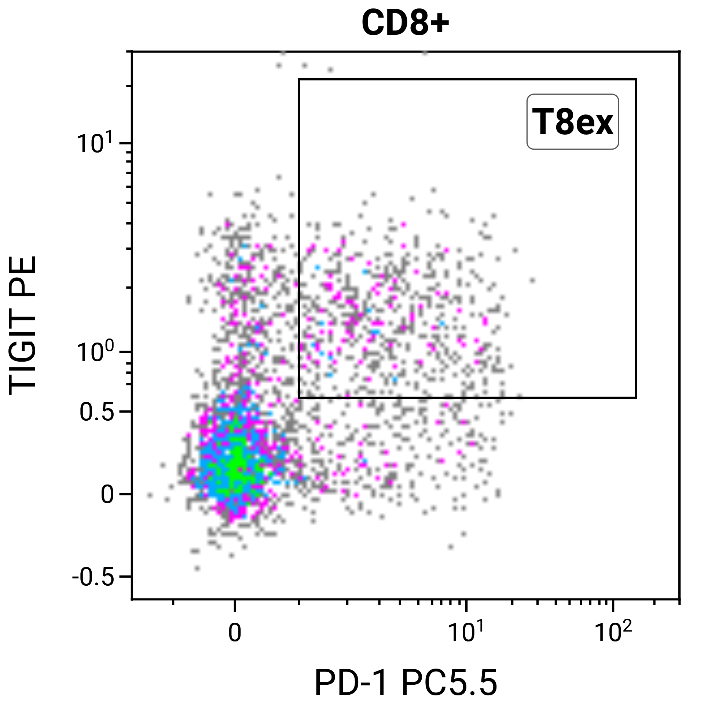


**Supplementary Figure 1**:

Gating strategy for CD4 *PD-1^+^ TIGIT^+^ and CD8^+^ T cells PD-1^+^ TIGIT^+^*identification using flow cytometry.

**Supplementary Figure 2:**

***CD4^+^ PD-1^+^ TIGIT^+^ and CD8^+^ PD-1^+^ TIGIT^+^* are not associated with disease activity.**

**A.** Proportion *CD4^+^ PD-1^+^ TIGIT^+^* depending on baseline disease activity (DAS28 for RA, ESSDAI for Sjögren, BASDAI for AS). **B.** Proportion of *CD8^+^ PD-1^+^ TIGIT^+^* depending on baseline disease activity (DAS28 for RA, ESSDAI for SjD, BASDAI for SPA).

*For RA patients, remission is defined as DAS28≤2.6, low disease activity as 2.6<DAS28≤3.2, moderate activity as 3.2<DAS28≤5.1 and high activity as 5.1>DAS28.*

*For Sjögren patients, low activity is defined as ESSDAI<5, moderate activity is defined as 5 ≤ ESSDAI<13, high activity is defined by ESSDAI≥13.*

*For SPA patients, low activity is defined by BASDAI<40 and high activity is defined as BASDAI≥40.*

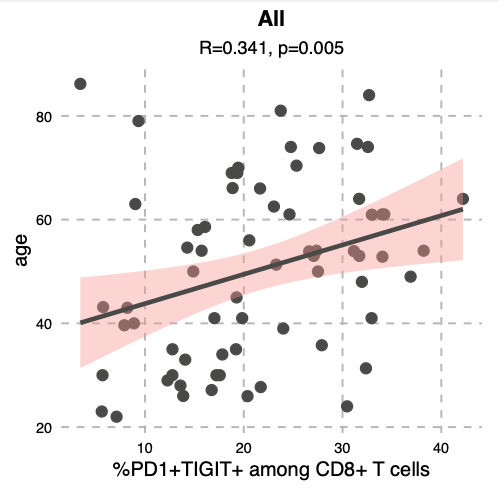


**Supplementary figure 3:** Correlation between age and CD8^+^ PD-1^+^ TIGIT^+^ cells in all patients (left) and in SPA patients (right)

**Supplementary table 3:**

Multivariate analysis using a linear regression model including age, sex and disease type.

|  | Term | Coefficient | %95CI lower bound | %95CI upper bound | Standard Error | p value |
| --- | --- | --- | --- | --- | --- | --- |
| CD4+ | Age | 0,058 | -0,026 | 0,143 | 0,042 | 0,175 |
|  | Sexe (male vs. female) | -0,038 | -3,495 | 3,419 | 1,735 | 0,983 |
|  | **IMID (SjD vs. RA)** | **5,302** | **2,091** | **8,513** | **1,611** | **0,002** |
|  | IMID (SPA vs. RA) | -0,596 | -4,445 | 3,254 | 1,931 | 0,759 |
| CD8+ | Age | 0,130 | -0,007 | 0,267 | 0,069 | 0,063 |
|  | Sexe (male vs. female) | -0,406 | -6,027 | 5,215 | 2,820 | 0,886 |
|  | IMID (SjD vs. RA) | -1,361 | -6,582 | 3,859 | 2,620 | 0,605 |
|  | IMID (SPA vs. RA) | -3,635 | -9,894 | 2,623 | 3,140 | 0,251 |

**Supplementary Figure 4:**

**PD-1^+^ TIGIT^+^ CD4^+^ cells association with serum inflammatory cytokines in IMIDs.**

Correlation plot of baseline circulating inflammatory cytokines and the proportion of PD-1^+^ TIGIT^+^ among CD4^+^ T cells in patients with RA (n=25), SPA (n=22), SjD (n=20), and in the overall cohort (n=67).

*ns : p>0.10, ° : p <0.10, * : p<0.05, ** : p<0.01, *** : p<0.001*

**Supplementary Figure 5:**

**CD8 PD-1+ TIGIT+ proportion is not associated with plasmatic cytokines concentration in patients with IMIDs.**

Correlation matrix between CD8 PD-1+ TIGIT+ proportion and plasmatic soluble factors for each IMID cohort.

*CRP : C-reactive protein. IMIDs : immune-mediated inflammatory diseases. SjD : Sjögren’s disease. RA : rheumatoid arthritis. SPA : spondylarthritis.*

*ns : p>0.10, ° : p <0.10, * : p<0.05, ** : p<0.01, *** : p<0.001*

**Supplementary Figure 6:**

Proportion of PD-1^+^ TIGIT^+^ among CD8^+^ T cells depending on response to anti-TNF treatment (n=7 non-responders, n=23 responders).

**Supplementary figure 7:**

Levels of baseline circulating cytokines and CRP according to response status in All patients (RA&SPA) and in individual diseases.

**Supplementary Figure 8**:

Comparison of the expression of CD4+, CD4+ PD-1+, CD4+TIGIT+and CD4+PD-1+ TIGIT+ at baseline in RA and SPA patients between future responders (Yes) and non- responders (No).

D

C

A

B

**Supplementary figure 9:**

ROC curves predicting response to anti-TNF treatment in RA and SPA patients of **A.**%CD4 T cells at baseline **B.** %CD4+PD-1+ T cells at baseline **C.** %CD4+TIGIT+ T cells at baseline **D.**% PD-1+TIGIT+ CD4 T cells among CD4 T cells at baseline
